# Epidemiology and costs of diabetes mellitus in Switzerland: an analysis of health care claims data, 2006 and 2011

**DOI:** 10.1186/1472-6823-14-44

**Published:** 2014-06-03

**Authors:** Carola A Huber, Matthias Schwenkglenks, Roland Rapold, Oliver Reich

**Affiliations:** 1 Department of Health Sciences, Helsana Insurance Group, P.O. Box, Zürich, Switzerland; 2 Institute of Social and Preventive Medicine, University of Zürich, 8001 Zürich, Switzerland

**Keywords:** Diabetes, Prevalence, Incidence, Mortality, Costs

## Abstract

**Background:**

Quantifying the burden of diabetes mellitus is fundamental for managing patients in health service delivery systems and improves the understanding of the importance of prevention and early intervention of diabetes. In Switzerland, epidemiological data on diabetes are very scarce. In this study we provide a first national overview of the current situation of diabetes mellitus in Switzerland as well as the development of the prevalence, incidence, mortality and costs between 2006 and 2011.

**Methods:**

Using health care claims data of a large health insurance group, current epidemiology and costs were determined from a sample of adult enrollees in 2011. The identification of patients with diabetes was based on prescription data of diabetes related drugs using the Anatomical Therapeutic Chemical Classification as proxy for clinical diagnosis. We further evaluated changes in epidemiology and costs between 2006 and 2011. All results were weighted with census data to achieve an extrapolation to the Swiss general population level.

**Results:**

A total of 920’402 patients were enrolled in 2011 and 49’757 (5.4%) were identified as diabetes cases. The extrapolated overall prevalence of diabetes in Switzerland was 4.9% (2006, 3.9%). The incidence was 0.58% in 2011 (2007, 0.63%). The extrapolated mortality rate was 2.6% with no significant change over time. Annual diabetes costs to the mandatory health insurance increased from EUR 5,036 per patient in 2006 to EUR 5’331 per patient in 2011.

**Conclusions:**

This study shows a high medical and economic burden of diabetes. The prevalence and costs of diabetes in Switzerland increased substantially over time. Findings stress the need for public health strategies to manage patients with chronic conditions and optimize resource allocation in health service delivery systems.

## Background

The management of patients with diabetes mellitus is a major challenge for health care systems. The International Diabetes Federation (IDF) estimates that about 366 million people suffered from diabetes worldwide in 2011, and the prevalence is expected to rise to 552 million by 2030 [1]. Diabetes is often associated with microvascular complications and macrovascular complications such as heart disease (heart attack, angina pectoris, heart failure) or hypertension [2,3], and leads to a significant burden of mortality [4,5]. Due to medical expenditures attributed to diabetes and its complications, as well as productivity losses of the patients, the economic cost of diabetes is immense [6,7]. The American Diabetes Association reported that the total estimated cost of diabetes was $174 billion in 2007 in the United States [8].

In Switzerland, epidemiological data providing information on the burden of diabetes are scarce. There are a few studies, which used different approaches to estimate the prevalence [9-11] or the direct costs of diabetes [12,13], and showed heterogeneous results. Furthermore, they were based on small study samples, not generalizable to the Swiss population, or are now outdated. Studies providing a current and comprehensive overview of the epidemiology and economic burden of diabetes are completely missing.

In absence of reliable population-based data on diabetes, health care claims data provide a useful basis for assessing the epidemiology and cost of diabetes. Claims data are reliable, longitudinal, cover a large population and are widely used in epidemiological, health services and outcome research e.g. [14].

The aim of the study was to provide at first a comprehensive and up-to-date overview of the Swiss situation. Using longitudinal data from a large health insurance population allowed us to assess the current state as well as potential changes in diabetes prevalence, incidence and mortality between 6 years. Moreover, we also aimed to determine the economic burden of diabetes including the total health care costs from a social insurance payer perspective.

## Methods

### Study design and study population

Health insurance is mandatory in Switzerland and the basic benefit package is the same in the entire country. The database underlying this study included mandatory health insurance claims from approximately 1.2 million persons which lived all over Switzerland and were enrolled with the Helsana Group, consisting of four health and accident insurance plans. In a first step, we defined a sample which included all subjects which were at least 19 years old in 2011 (2011 sample; n = 1’015’106). These data were used to give information about the recent epidemiology and costs of diabetes in Switzerland. In order to additionally identify changes since 2006, we defined a further sample including all persons aged over 19 years in 2006 (2006 sample; n = 920’402). For prevalence and costs estimates, both the 2006 and 2011 samples included patients who were alive and continuously enrolled in the given year. For mortality estimates, we included all individuals. In order to determine the incidence of diabetes, we defined in a next step a retrospective cohort (2006–2011) which included adult persons who were continuously enrolled with Helsana Group during the 6-year period, from January 1, 2006, until December 31, 2011. Subjects who died within this period were also included. This cohort included 477’870 persons at risk in 2006 and 3’993 persons of those were new diabetes cases in 2007 (total: n = 491’863). Available population characteristics included gender, age, language area and type of insurance coverage (managed care model, deductible class). Analyzed data also contained information on patients’ treatment and its associated health care cost; they covered all settings of outpatient and inpatient care and nursing. Since the recorded insurance claims cover almost all health care invoices, the data achieve a high degree of completeness.

According to the national ethical and legal regulation, an ethical approval was not needed. Permission to access the study data was provided by the Helsana Group.

### Identification of patients with diabetes mellitus

In absence of epidemiological data including medical diagnoses (e.g. International Classification System, ICD), the WHO Anatomical therapeutic chemical (ATC) [15] classification system was used to identify patients with diabetes. Patients were classified as having diabetes when they were prescribed at least one drug for diabetes (hypoglycemic medication or insulin; ATC code A10A), oral blood glucose lowering drug (ATC code A10B) or other drug used in diabetes (ATC code A10X) anytime in a given calendar year. Patients with a prescription for diabetes in a given year were considered as still prevalent in the following year, irrespective of whether were additional prescriptions. This mapping approach allows a direct measure of treated diabetes and is frequently used as a reliable way to identify chronically ill persons in administrative data samples [e.g.16]. Based on previous studies, the use of at least one prescription for diabetes drugs can be used for an accurate identification of patients with diabetes across a large population e.g. [17].

### Estimation of prevalence, incidence and mortality

Using our diabetes classification, we calculated diabetes prevalence for the 2006 and 2011 samples. The number of patients with diabetes was divided by all insured persons in the sample in the same year. Prevalence rates were stratified by age and sex. Furthermore, we used census data from the Swiss Federal Office of Statistics to extrapolate the prevalence of patients with diabetes in the general adult population for each year. The characteristics of the of the study population and the entire Swiss population are shown in Table 1. We assumed that our study sample is approximately representative. However, the sample included a slightly higher proportion of elderly persons and the market share of Helsana is not equally distributed in all 26 Swiss cantons. Therefore we used the method of extrapolation. We construct various levels in which the insured persons are represented and weighted the population by the levels which included the entire Swiss population (census data).The stratification for the extrapolation was carried out by age, gender and canton of residence. All 26 Swiss cantons and a total of 16 age classes, which are based on the classification of the risk equalization statistics, were taken into account [18].

**Table 1:** Characteristics of the study population and the entire Swiss population

	**Study population (2011)**		Entire Swiss population (2011)^*^	
	**N**	**%**	**N**	**%**
Overall				
All ages	920402		6’407’133	
19-39	282742	30.72	2’215’128	34.57
40-59	315446	34.27	2’369’500	36.98
>59	322214	35.01	1’822’505	28.44
Women				
All ages	481616	52.33	3’279’256	51.18
19-39	138936	28.85	1’097’171	33.46
40-59	157528	32.71	1’175’379	35.84
>59	185152	38.44	1’006’706	30.70
Men				
All ages	438786	47.67	3’127’877	48.82
19-39	143806	32.77	1’117’957	35.74
40-59	157918	35.99	1’194’121	38.18
>59	137062	31.24	815’799	26.08

^*^according to the census data of the Swiss Federal Office of Statistic [19].

The annual incidence of diabetes was defined as the number of newly identified cases with diabetes divided by the study population which is free of diabetes at the beginning of the given year. Our calculations excluded the number of persons with prevalent diabetes in the first study year. We classified patients as incident cases if they were identified as persons with diabetes in a given year and had a minimum diabetes-free period of one year in the previous years. Incidence estimates were based on the cohort data (2006–2011 cohort) and additionally standardized to the Swiss population using census data.

We included mortality as a further outcome variable. All deaths of the study sample in 2006 and 2011 were recorded but there was no information on cause of death. Therefore, we calculated the annual all-cause mortality in patients with diabetes, and additionally extrapolated to the general Swiss population by census data.

### Estimation of costs

The analysis investigating the health care costs of diabetes was conducted from the perspective of the Swiss statutory health insurance system. Annual total health care costs (Swiss Francs (CHF) exchanged to Euro (EUR); rate of 0.81) of diabetes patients were obtained from providers’ claims and defined as the total payments made by the mandatory health insurance for outpatient and inpatient services per patient and year. Costs from the outpatient setting comprised payments for office-based physician visits, hospital outpatient visits, paramedical visits, nursing home, medications, laboratory tests and medical devices. Inpatient costs covered payments for hospital treatments, hospitalizations, rehabilitation, nursing home and emergency transport services. Inpatient costs also cover the cost of medications, laboratory and medical services during the inpatient episode.

Mean annualized costs of diabetes patients including standard deviation were calculated for each age and sex group. Furthermore, we estimated the costs attributable to diabetes by calculating the difference between the mean costs per capita/year in patients with diabetes and the mean cost per capita/year in patients without diabetes. Estimations were based on the 2006 and 2011 samples.

Additionally, we used census data to extrapolate the health care costs of patients with diabetes to the general adult population for each year.

### Estimation of time trends

To determine time trends in epidemiology and costs, we additionally estimated logistic regression models using year as a binary variable for comparisons of prevalence, mortality and costs, based on insured persons in the 2006 and 2011 samples. Year was treated as a continuous variable for determining time trends in incidence in the 2006–2011 cohort sample. Estimates were reported as Odds Ratios (OR) including associated 95% confidence interval (95%-CI). All regression models were controlled for age and sex. Given the skewed nature of costs, we used a multiple regression model with log-transformed costs as outcome. Results were given as exponentiated coefficients with 95%-CI. We reported p-values <0.05 as significant. All statistical analyses performed using R, version 2.14.2. For performing the extrapolation the R-package “survey” was used.

## Results

Our study population included 920’402 subjects comprising about 50.000 patients with diabetes mellitus in 2011 (Table 2).

**Table 2:** Age- and sex specific prevalence of diabetes in 2006 and 2011

**Prevalence**									
	**2006**				**2011**				
**Group**	**No. with diabetes**	**Population without diabetes**	**Prevalence rate (%) unadjusted**	**Prevalence rate (%, 95–CI**^**a**^**) extrapolated**^**b**^	**No. with diabetes**	**Population without diabetes**	**Prevalence rate (%) unadjusted**	**Prevalence rate (%, 95–CI**^**a**^**) extrapolated**^**b**^	**Difference**^**c**^
Overall									
All ages	41’963	973’143	4.13	3.89 (3.87–3.93)	49’757	870’645	5.41	4.90 (4.86–4.94)	1.01
19–39	1’794	342’167	0.52	0.54 (0.52–0.56)	1’918	280’824	0.68	0.69 (0.65–0.73)	0.15
40–59	9’725	339’452	2.79	2.80 (2.74–2.86)	11’176	304’270	3.54	3.62 (3.54–3.70)	0.82
>59	30’444	291’524	9.46	9.63 (9.53–9.73)	36’663	285’551	11.38	11.68 (11.56–11.80)	2.05
Women									
All ages	19’609	513’284	3.68	3.37 (3.33–3.41)	22’724	458’892	4.72	4.15 (4.09–4.21)	0.78
19–39	977	167’320	0.58	0.59 (0.55–0.63)	1’094	137’842	0.79	0.79 (0.73–0.85)	0.20
40–59	3’779	173’579	2.13	2.12 (2.06–2.18)	4’419	153’109	2.81	2.79 (2.71–2.87)	0.67
>59	14’853	172’385	7.93	7.98 (7.86–8.10)	17’211	167’941	9.30	9.41 (9.27–9.55)	1.43
Men									
All ages	22’354	459’859	4.64	4.45 (4.39–4.51)	27’033	411’753	6.16	5.68 (5.62–5.74)	1.23
19–39	817	174’847	0.47	0.48 (0.43–0.51)	824	142’982	0.57	0.58 (0.54–0.62)	0.10
40–59	5’946	165’873	3.46	3.48 (3.40–3.56)	6’757	151’161	4.28	4.44 (4.32–4.56)	0.96
>59	15’591	119’139	11.57	11.74 (11.56–11.92)	19’452	117’610	14.19	14.49 (14.29–14.69)	2.75

^a^95% Confidence Interval.

^b^Prevalences are extrapolated to the Swiss general population in 2006 and 2011.

^c^Difference between the extrapolated prevalence rates in 2006 and 2011.

### Prevalence

Table 2 shows the overall prevalence of diabetes of 5.4%. Extrapolated to the adult population of Switzerland, the overall prevalence of diabetes was 4.9% in 2011. The number of patients with diabetes increased with age in both men and women. The extrapolated overall prevalence was higher in men than in women [5.7% (CI: 5.6-6.7) versus 4.2% (CI: 4.1-4.2)] in 2011. However, in the younger age group (19–39 years), women had a higher prevalence rate than men [0.8% (CI: 0.7-0.9) vs. 0.6% (CI: 0.5-0.6)]. In the unadjusted as well as in the extrapolated 2011-sample, all prevalence rates in each subgroups (sex/age) showed the same trends, but at a higher level in the unadjusted sample.

The overall prevalence of diabetes increased from 3.9% in 2006 to 4.9% in 2011. Moreover, the multivariable regression model showed a significant increase of the diabetes prevalence between 2006 and 2011 (OR 1.24, 95%-CI: 1.22-1.26; Table 3).

**Table 3:** Time trend and change in prevalence, incidence, mortality and costs (2006–2011)

	**OR (95%-CI)**^**a**^
**Outcomes**	**Logistic regression model**^**b**^
Prevalence:	1.24 (1.22-1.26)^*^
Time change (2006/ 2011)	
Incidence^c^:	0.96 (0.95-0.97)*
Time trend (1-year increase)	
Mortality^d^:	1.07 (0.99-1.16)
Time change (2006/ 2011)	
	**Exp(coefficient)**
	**Linear regression model with log-transformed costs**^**b**^
Costs^c^:	1.08 (1.06-1.09^)*^
Time change (2006 to 2011)	

^a^Odds ratio with 95% confidence intervals.

^b^Controlled for age and sex.

^c^Time trend over the 6-year period 2006–2011.

^d^Among persons with diabetes.

^*^p-value <0.05.

### Incidence

All incidence estimates presented in Table 4 showed similar trends both in the unadjusted and the extrapolated sample. The unadjusted overall incidence of diabetes was 0.7%, with a higher rate in men than in women in 2011 (0.9% vs. 0.7%; Table 4). The extrapolated incidence of diabetes in 2011 was overall 0.6%, and 0.7% in men and 0.5% in women. The extrapolated incidence was highest in men aged over 59 years (1.4%).

**Table 4:** Age- and sex specific incidence of diabetes in 2007 and 2011

**Incidence**									
	**2007**				**2011**				
**Group**	**No. with new diabetes**	**Population at risk (2006)**	**Incidence (%) unadjusted**	**Incidence (%, 95% CI**^**a**^**) extrapolated**^**b**^	**No. with new diabetes**	**Population at risk (2010)**	**Incidence (%) unadjusted**	**Incidence (%, 95% CI**^**a**^**) extrapolated**^**b**^	**Difference**^**c**^
Overall									
All ages	3’993	477’870	0.84	0.63 (0.61–0.65)	3’301	447’390	0.74	0.58 (0.56–0.60)	−0.05
19–39	149	88’833	0.17	0.16 (0.13–0.18)	119	85’270	0.14	0.17 (0.14–0.20)	0.01
40–59	1’009	161’989	0.62	0.61 (0.57–0.65)	812	152’714	0.53	0.56 (0.52–0.60)	−0.05
>59	2’835	227’048	1.25	1.26 (1.21–1.31)	2’300	223’395	1.03	1.10 (1.05–1.15)	−0.16
Women									
All ages	1’918	264’173	0.73	0.54 (0.51–0.57)	1’595	246’612	0.65	0.51 (0.48–0.55)	−0.03
19–39	96	42’879	0.22	0.20 (0.16–0.25)	96	39’872	0.24	0.25 (0.20–0.30)	0.05
40–59	425	84’330	0.50	0.47 (0.42–0.53)	351	74’740	0.47	0.45 (0.44–0.50)	−0.02
>59	1’397	136’964	1.02	1.01 (0.95–1.06)	1’148	132’000	0.87	0.87 (0.82–0.93)	−0.14
Men									
All ages	2’075	213’697	0.97	0.73 (0.69–0.77)	1’706	200’778	0.85	0.65 (0.61–0.69)	−0.08
19–39	53	45’954	0.12	0.11 ()0.08–0.14	39	44’134	0.09	0.09 (0.06–0.12)	−0.02
40–59	584	77’659	0.75	0.75 (0.67–0.82)	507	69’311	0.73	0.67 (0.60–0.73)	−0.08
>59	1’438	90’084	1.60	1.58 (1.50–1.67)	1’160	87’333	1.33	1.39 (1.30–1.47)	−0.19

^a^95% Confidence Interval.

^b^Incidences are extrapolated to the Swiss population in 2007 and 2011.

^c^Difference between the extrapolated incidences 2007 and 2011.

Overall, the incidence of diabetes decreased from 0.8% in 2006 to 0.7% in 2011, representing a relative change of 12%. The annual relative decrease in incidence was about 4.0% (OR 0.96, 95% CI: 0.95-0.97; Table 3). Decrease in incidence was similar in men and in women (change of 11% resp. 12%) and in persons aged >59 years compared to those aged 40–59 years (change of 18% vs. 15%).

### Mortality

Estimations based on the 2006 and 2011 sample were based on 43’444 and 51’547 patients with diabetes, respectively (Table 5).

**Table 5:** Age- and sex specific mortality rate in prevalent diabetes cases in 2006 and 2011

**Mortality**									
	**2006**				**2011**				
**Group**	**No. of deaths**^**a**^	**Population with diabetes**	**Mortality rate (%) unadjusted**	**Mortality rate (%, 95% CI**^**b**^**) extrapolated**^**c**^	**No. of deaths**^**a**^	**Population with diabetes**	**Mortality rate (%) unadjusted**	**Mortality rate (%, 95% CI**^**b**^**) extrapolated**^**c**^	**Difference**^**d**^
Overall									
All ages	1’148	43’444	2.64	2.49 (2.35–2.63)	1’481	51’547	2.87	2.56 (2.42–2.70)	0.07
19–39	4	1’862	0.21	0.30 (0.02–0.58)	3	1’993	0.15	0.14 (−0.02–0.30)	−0.16
40–59	70	9’995	0.70	0.67 (0.51–0.83)	75	11’424	0.66	0.68 (0.52–0.84)	0.01
>59	1’074	31’587	3.40	3.35 (3.15–3.55)	1’403	38’130	3.68	3.46 (3.28–3.64)	0.11
Women									
All ages	525	20’239	2.59	2.50 (2.28–2.72)	670	23’538	2.85	2.53 (2.33–2.73)	0.03
19–39	2	1’011	0.20	0.27 (−0.09–0.63)	2	1’126	0.18	0.16 (−0.06–0.38)	−0.11
40–59	23	3’866	0.59	0.60 (0.36–0.84)	21	4’518	0.46	0.49 (0.27–0.71)	−0.11
>59	500	15’362	3.25	3.27 (2.99–3.55)	647	17’894	3.62	3.44 (3.30–3.58)	0.17
Men									
All ages	623	23’205	2.68	2.49 (2.29–2.69)	811	28’009	2.90	2.57 (2.39–2.75)	0.08
19–39	2	851	0.24	0.34 (−0.12–0.80)	1	867	0.12	0.12 (−0.10–0.34)	−0.22
40–59	47	6’129	0.77	0.72 (0.52–0.92)	54	6’906	0.78	0.80 (0.58–1.02)	0.08
>59	574	16’225	3.54	3.41 (3.13–3.69)	756	20’236	3.74	3.48 (3.24–3.72)	0.07

^a^Among persons with diabetes.

^b^95% Confidence Interval.

^c^Mortality rates are extrapolated to the Swiss population in 2006 and 2011.

^d^Difference between the extrapolated mortality rates 2006 and 2011.

The unadjusted annual all-cause mortality in patients with diabetes in 2011 was 2.9%, and the extrapolated mortality 2.6%, with no significant differences between the sexes [2.5% (CI: 2.3-2.7) vs. 2.5% (CI: 2.4-2.8), Table 5]. As with prevalence and incidence, mortality was higher in older persons than in younger persons in the unadjusted as well as in the extrapolated sample.

The extrapolated all-cause mortality rate slightly increased from 2.5% in 2006 to 2.6% in 2011. However, the logistic regression model showed no significant increase in mortality between 2006 and 2011 (OR 1.07, 95% CI: 0.99-1.16; Table 3).

### Costs

As presented in Table 6, annual total health care costs (Euro (EUR)) of diabetes patients showed similar trends both in the unadjusted and the extrapolated sample. Overall, diabetes patients accrued total health care costs of EUR 8’424 (unadjusted) and EUR 8’239 (extrapolated), respectively, per patient and year in 2011. Extrapolated mean costs in women were higher than in men (EUR 8’679 vs. EUR 7’902) and highest in patients aged over 59 years (EUR 8’993).

**Table 6:** Age- and sex specific mean costs in patients with and without diabetes 2006 and 2011

**Costs**										
	**2006**				**2011**					
**Group**	**Patients with diabetes**		**Patients without diabetes**		**Patients with diabetes**			**Patients without diabetes**		
	**Mean costs (EUR) unadjusted**	**Mean costs (EUR) extrapolated**^**a**^	**Mean costs (EUR) unadjusted**	**Mean costs (EUR) extrapolated**^**a**^	**Costs attributable to diabetes**^**b**^	**Mean costs (EUR) unadjusted**	**Mean costs (EUR) extrapolated**^**a**^	**Mean costs (EUR) unadjusted**	**Mean costs (EUR) extrapolated**^**a**^	**Costs attributable to diabetes**^**b**^
Overall										
All ages	7489	7495	2564	2459	5036	8424	8239	3157	2907	5331
19–39	6350	6533	1264	1293	5241	6589	6798	1567	1567	5231
40–59	6226	6264	1963	1967	4296	6703	6613	2349	2389	4224
>59	7960	8038	4790	4799	3239	9048	8993	5583	5471	3522
Women										
All ages	7916	7957	3008	2882	5075	8837	8679	3699	3402	5277
19–39	6304	6504	1665	1693	4811	6974	7238	2074	2066	5173
40–59	6764	6853	2219	2234	4619	7110	7079	2672	2717	4363
>59	8319	8424	5105	5147	3277	9404	9361	5971	5851	3510
Men										
All ages	7116	7121	2069	2003	5118	8078	7902	2553	2381	5521
19–39	6404	6569	880	893	5676	6077	6207	1078	1080	5127
40–59	5885	5909	1697	1700	4209	6437	6325	2023	2061	4265
>59	7622	7701	4332	4334	3367	8732	8697	5030	4973	3724

^a^Mean costs are extrapolated to the Swiss population in 2006 and 2011.

^b^Difference between the extrapolated mean costs in 2006 and 2011.

The extrapolated annual costs attributable to diabetes (i.e. difference in mean costs between insured persons with vs. without diabetes) were EUR 5’331 in 2011, with higher costs in men than in woman (EUR 5’521 vs. EUR 5’277). The lowest costs were observed in the age group >59 years, in both sexes.

Overall, the costs attributable to diabetes increased from EUR 5’036 in 2006 to EUR 5’331 in 2011. Regression analysis showed a significant increase in costs among patients with diabetes between 2006 and 2011 (coefficient 1.08, 95% CI: 1.06-1.09; Table 3).

## Discussion

This study is the first national study to provide a comprehensive overview of the burden of diabetes mellitus in Switzerland. Based on a large data set, we estimated the current state as well as the development in diabetes prevalence, incidence, mortality and cost-of-illness between 2006 and 2011.

This study yielded an population prevalence of 4.9% in 2011. Our finding is lower than the prevalence rate of the Swiss CoLaus study (6%) of subjects aged 35–75 years and living in Lausanne in 2003 [9]. However, prevalence results originating from other studies performed in Switzerland were significantly lower than our result which might be explained by variations in study design and definition of diagnosed diabetes. For example, Bopp et al. [10] analyzed diagnoses of hospital discharges and the death registry and reported a diabetes prevalence of about 3.5-4.3% in 2007/2008. This finding might be an underestimation, since diabetes is often not the primary reason for hospitalization and thus not stated in all medical records. Our estimates of prevalence and upward time trend are more in line with international studies reporting higher prevalence levels in the general population. For example, Wilke et al. [20] reported a standardized prevalence of 5.5% for patients with Type 2 diabetes of all ages in Germany in 2008. Additionally, we observed a significant increase in diabetes prevalence between 2006 and 2011. This increasing trend is in line with estimates from Germany [6] and Canada [5] and from global estimates of the IDF Atlas (International Diabetes Federation), which predicted a major increase of 51% in worldwide cases with diabetes by 2030 [1].

While the diabetes prevalence in the Swiss population is increasing, our study suggests a decreasing incidence of treated diabetes over the 6-year time period. The extrapolated incidence of diabetes showed a relative decrease by 8% (percentage change from 2007 to 2011). Previous studies performed in Canada, U.S. and Italy, examining trends in diabetes incidence provide conflicting results [5,21,22]. Differences in incidence trends of diabetes might be associated with several risk factors such as lifestyle habits (i.e. smoking), overweight and environmental patterns which vary between countries and cultures. Since obesity as a strong risk factor for diabetes is rising in Switzerland [23], the observed decrease in diabetes incidence suggests a meaningful improvement in other diabetes related risk factors. Previous research has shown the positive impact of lifestyle interventions (exercise and/or diet) to reduce the diabetes incidence and indicates that the effectiveness of the intervention is independent from weight-changes [24]. However, time-point incidence rates of most previous studies corroborate the results of our study, where the estimated diabetes incidence was 0.5-0.9%.

Our all-cause mortality estimates for diabetes patients are higher than those found in other studies [5,25,26]. For example, findings from Canada reported a decrease of 3.3 to 1.6 in all-cause mortality from 1995 to 2005 [5]. However, comparability is limited since considered time intervals differ from each other.

In this study, we also determined the mean annual direct medical costs of diabetes. Patients with diabetes caused health care costs of EUR 8‘239 per year; comparison with persons without diabetes lead to a total of EUR 5’331 per patient attributable to diabetes in 2011. Our cost estimates from 2011 suggest a meaningful increase in direct costs (excluding nursing home), when comparing with previous national estimates in patients with type 2 diabetes from 1998/1999 (EUR 2’232) [12]. Comparison with international results is difficult as health systems, medical practice and unit cost differ greatly. Köster et al. [6] showed that the average health care costs (including nursing home) of patients with diabetes in Germany were EUR 5’726 in 2007 and that the costs attributable to diabetes were EUR 2’605. Another study calculating the medical costs of diabetes from the U.S. reported an annual cost (including nursing care) per diabetes case of EUR 5’120 in 2007 [27], which is meaningful lower than findings from our study. Additionally, our results show an increase of the cost attributable to diabetes in 2006 to 2011. This finding is in line with internationally reported data showing an increase of health care costs over the past years [28,29]. According to our results, about 387’100 persons (4.9% of 8 million Swiss residents) were diagnosed with diabetes and treated in 2011. This implies total health care costs attributable for diabetes in the compulsory health insurance of CHF 2.5 billion, corresponding to 9.5% of total Swiss statutory health insurance expenditures in 2011. This finding represents a significant burden of disease in Switzerland, and is expected to rise in the future, due to demographic changes as increasing life expectancy and increasing number of elderly patients [30]. In addition, with the current increase in overweight and obesity observed in Switzerland [23], it stresses the need for public health strategies to manage patients with diabetes and optimize resource allocations in health service delivery systems. The promotion of chronic care management might be an effective approach to manage and improve health care for patients with diabetes [31] and adequately prepare for the increasing burden of diabetes on health care resources. Moreover, our results highlight the importance of prevention and early intervention of diabetes mellitus, especially in the elderly population. There is a meaningful impact of age on the occurrence of diabetes mellitus. The percentage of elderly persons continues to rise and older people are faced with chronic diseases and multimorbidity. Previous research has shown that lifestyle interventions are very effective in preventing or delaying the occurrence of chronic diseases such as diabetes mellitus [23,32]. Physical activity and healthy diet helps to prevent diabetes (type 2). Public health efforts should notably address the prevention of diabetes in the elderly population and give it a priority in health policy, decision-making and resource allocation debates.

Several strengths and limitations of our study have to be taken into account. The main strength is that the study is based on a very comprehensive health care claims database which covers a large and geographically diverse Swiss population and allows for a good and low cost monitoring of diabetes occurrence. Since we additionally performed an extrapolation of our estimates to the general adult population, we regard our results as being representative for the prevalence, incidence, mortality and cost of diabetes for Switzerland. The study also has several limitations. First, the number of patients with diabetes may be biased, because clinical diagnoses (e.g., ICD-10) were not available. Additionally, our prevalence might be underestimated. Undiagnosed persons with diabetes, patients with lifestyle-treatment and patients with diabetes who were non-adherent to their medication could not be included in the study. However, diagnoses based on dispended medications are a valid proxy for medical diagnoses and widely used in epidemiological and outcomes research to assess prevalence [14,16]. Second, cost estimates may be slightly too low as about 3% of the used claims were not paid by the health insurer but directly by the patient (out-of-pocket). Third, our cost estimates might be slightly overestimated, since the data do not allow a distinction between the costs of the treatment of diabetes solely and the costs of its major comorbidities. Further research is needed to estimate the costs of diabetes considering the occurrence of diabetes specific complications such as cardiovascular diseases. A fourth limitation of this study is that incidence trend estimations are not completely representative of the general population, because our cohort only included continuously enrolled patients; persons newly insured during the observation period were not covered. Since we have more or less high fluctuations in our stock of insured persons over the years, we assumed to have a switching bias in calculating the incidence. However, to define a consistent cohort of persons and to follow them over several years it is a very common and frequently used method to determine trends in diseases. Cohort studies are very important in giving us a lot of epidemiological information. Using this study design allows us to see what happens to them with diabetes as well as to see who will develop a new disease. We are able to follow the patients and thus to assess the incidence and its trend.

## Conclusions

In conclusion, this study shows a high medical and economic impact of diabetes mellitus. The prevalence and costs of diabetes in Switzerland increased substantially between 2006 and 2011. These findings highlight the need for improvements in prevention and early intervention of diabetes mellitus. Effective strategies should be developed to manage patients with chronic conditions and to optimize resource allocations in health service delivery systems of those with diabetes.

## Competing interests

Financial support for this study was provided by Merck, Sharp and Dohme & Co (MSD), Switzerland. The sponsor had no role in collection, analysis, and interpretation of data; writing of the paper; or in the decision to submit the paper for publication. MS received no funding.

## Authors’ contributions

CAH, MS and OR performed the conceptual development and the study design. CAH and RR analyzed the data and CAH drafted the manuscript. OR and MS revised the manuscript. All authors participate in the interpretation of data, critically reviewed for important intellectual contents and gave the final approval of the version to be published.

## Pre-publication history

The pre-publication history for this paper can be accessed here:

http://www.biomedcentral.com/1472-6823/14/44/prepub
